# Influence of Ultrafine-Grained Microstructure and Texture Evolution of ECAPed ZK30 Magnesium Alloy on the Corrosion Behavior in Different Corrosive Agents

**DOI:** 10.3390/ma15165515

**Published:** 2022-08-11

**Authors:** Abdulrahman I. Alateyah, Majed O. Alawad, Talal A. Aljohani, Waleed H. El-Garaihy

**Affiliations:** 1 Department of Mechanical Engineering, College of Engineering, Qassim University, Unaizah 56452, Saudi Arabia; 2 Materials Science Research Institute, King Abdulaziz City for Science and Technology (KACST), Riyadh 12354, Saudi Arabia; 3 Mechanical Engineering Department, Faculty of Engineering, Suez Canal University, Ismailia 41522, Egypt

**Keywords:** equal channel angular pressing, sever plastic deformation, ultrafine-grained structure, crystallographic texture, corrosion behavior, potentiodynamic polarization, electrochemical impedance spectroscopy

## Abstract

Magnesium-Zinc-Zirconium (Mg-Zn-Zr) alloys have caught considerable attention in medical applications where biodegradability is critical. The combination of their good biocompatibility, improved strength, and low cytotoxicity makes them great candidates for medical implants. This research investigation is focused on providing further insight into the effects of equal channel angular processing (ECAP) on the corrosion behavior, microstructure evolution, and mechanical properties of a biodegradable ZK30 alloy. Billets of Mg-3Zn-0.6 Zr (ZK30) alloy were processed through ECAP up to 4 passes of route Bc (rotating the billets 90° in the same direction between the subsequent passes) at 250 °C. Electron back-scatter diffraction (EBSD) was utilized to investigate the microstructural evolution as well as the crystallographic texture. Several electrochemical measurements were carried out on both a simulated body fluid and a 3.5% sodium chloride (NaCl) solution. Mechanical properties such as Vicker’s hardness and tensile properties were also assessed. The as-annealed (AA) microstructure was dominated by equiaxed coarse recrystallized grains with an average grain size of 26.69 µm. After processing, a geometric grain subdivision took place due to the severe plastic deformation. Processed samples were characterized by grain refinement and high density of substructures. The 4-passes sample experienced a reduction in the grain size by 92.8% compared with its AA counterpart. The fraction of high-angle grain boundaries increased significantly after 4-passes compared to the 1-pass processed sample. With regards to the crystallographic texture, the AA condition had its {0001} basal planes mostly oriented parallel to the transversal direction. On the other hand, ECAP processing resulted in crystallographic texture changes, such as the shifting of the ZK30 shear plane to be aligned at 45° relative to the extrusion direction (ED). Furthermore, the maximum texture intensity was reduced from 14 times random (AA billets) to 8 times random after ECAP processing through 4-passes. The corrosion rate of the 4-passes sample was tremendously reduced by 99% and 45.25% compared with its AA counterpart in the simulated body fluid and the NaCl solution, respectively. The pitting corrosion resistance of ZK30 showed notable improvements in the simulated body fluid by 471.66% and 352% during processing through 1-pass and 4-passes, respectively, compared with the 3.5% NaCl findings. Finally, significant improvements in the tensile strength, hardness, and ductility were also achieved.

## 1. Introduction

Mg alloys have recently demonstrated a lot of potential for usage in the production of orthopedic implants that are biocompatible, biodegradable, and possibly bioactive. The presence of this combination of properties in Mg alloys makes the possibility of them replacing currently used materials, such as stainless steel, titanium alloys, and cobalt-chromium alloys, very feasible on account of these previous materials causing allergies and hypersensitivity [1,2,3,4,5,6]. Being the lightest metallic alloys, Mg alloys have mechanical properties comparable to that of human bone [7]. Furthermore, Mg alloys’ biodegradability in the human body, as they fade after the second surgery, is their most prominent advantage compared with other metallic alloys [7,8]. Particularly, Mg alloys have a density range from 1.75 up to 1.85 g/cm^3^ which mirrors the density range of human bones (1.75–2 g/cm^3^) [9]. Moreover, Mg alloys possess a modulus of elasticity of (41–45 GPa), that is more compatible with the human bone modulus (0.01–3.0 GPa) than the currently used medical metals, which leads to less stresses at the bone/implant interface [9,10].

Mg-based biodegradable substrates can be classified into four main groups: pure Mg, Mg-aluminum-containing alloys, Mg alloys containing rare earth elements, and aluminum-free alloys [11]. Amongst the aluminum-free Mg alloys, Mg-Zn alloys are renowned for being promising biodegradable materials. This is due to the strengthening effect Zn has on the alloy [1,5]. Zn is necessary for the progress of many biological functions and is naturally found in human muscles and bones as it plays a vital role in bone formation [9,12]. The addition of Zn with quantities up to 3%wt in Mg-Zn alloys leads to an increase in their tensile properties and corrosion resistance due to the formation of the heterogeneous second-phase particles (MgZn2) [1,13,14,15]. In addition, Zn provides a benefit, not only by refining the grain size, but also by playing a crucial role of age hardening, solid solution strengthening, and sterilization [16]. In particular, Mg-Zn-Zr or ZKxx alloys (Z for zinc and K for zirconium) have acquired great attention in degradable medical applications because of their good biocompatibility, adequate strength, and low cytotoxicity due to the absence of toxic elements such as aluminum [1,3,5,13]. The improved strength and corrosion resistance of Mg-Zn-Zr over Mg-Zn can be attributed to the grain refinement process promoted by Zr [17].

In bone repair medical applications, the implant material must exist un-degraded throughout the bone’s healing interval to perform its biochemical role [1]. Unfortunately, Mg alloys rapidly corrode before the necessary healing time and deteriorate, and their mechanical properties degrade [5]. Although solid solution strengthening with Zn and Zr significantly boosts Mg’s corrosion resistance, further enhancement through grain refinement proves necessary for the biodegradable alloys to accomplish their purpose adequately. Plenty of previous research has shown that grain refinement enhances metallic materials’ strength and corrosion properties [5,18,19,20,21]. Recently, SPD has been considered an efficient tool for the grain refinement of metallic materials because it can result in a homogeneous distribution of the nano-sized second phases particles [5,18,19,22,23,24]. Valiev et al. were the first to suggest using severe plastic deformation (SPD) in the processing of biomedical implants [25]. Various SPD techniques have been developed for producing ultra-fine grained (UFG) materials, such as equal channel angular pressing (ECAP) [26,27,28,29,30,31], high pressure torsion (HPT) [32,33,34], twist extrusion [35], accumulative rolling bonding [36], and multi-channel spiral twist extrusion (MCSTE) [37,38,39,40]. Among the different SPD techniques, ECAP has been proven to be the most potent technique in producing UFG or even nano-structured (NS) metallic materials. UFG or nano structures of ZKxx alloys translate into enhanced mechanical properties and corrosion resistance, which are much needed [23,26,41,42,43]. Other studies were conducted on the effect of plastic deformation on corrosion resistance. Notable attention was paid in these studies to the effect of grain refining, yielded from ECAP processing. One study showed that after investigating ZKxx alloys in a chloride environment, the alloys’ corrosion resistance had remarkably improved after ECAP processing. This resulted from the grain refinement and the subsequent more homogeneous distribution of Zn and Zr-alloying elements from using ECAP [13].

Besides the microstructure and the second phases, the corrosive agent the metallic material is subjected to affects its corrosion properties [43]. Until now, the bulk of research on Mg alloys’ corrosion resistance has been focused on how it is affected by grain refinement through ECAP. Some studies have used a NaCl solution as a corrosive agent to investigate the corrosion behavior of Mg alloys, such as AZ91 [22,30,44,45], AZ31 [46], ZK60 [43], Mg-Zn alloys [23,47], and rare-earth Mg alloys [48,49]. Other studies used a simulated body fluid to investigate Mg alloys, such as Mg-Zn-Zr [5], AZ31 [19,50], ZK60 [24], and ZN20 [51]. In addition, Hanks’ solution was used in some studies on Mg alloys, such as Mg-Zn-Ca [52,53], Mg-HA [54], Mg- ZKQX6000 [55], and ME21 [56], whereas Sigma-Aldrich R8758 solution was also used on the AZ31 alloy [57].

In light of the aforementioned literature, to the best of the authors’ knowledge, no body of work has previously reported on the effect the corrosion environment has on ECAPed Mg alloys. Furthermore, to date, investigations of the corrosion behavior of biodegradable ZK30 (Mg-3Zn-0.6 Zr, wt%) alloys after ECAP processing are insufficient. This current work is focused on providing further insight into the effect of ECAP on the corrosion resistance of biodegradable Mg ZK30 alloys. To that end, the aim of this paper is two-fold. Firstly, to understand the performance of the ZK30 alloy more thoroughly in aqueous corrosive media. This goal was achieved by investigating the corrosion behavior of the ZK30 Mg alloy in ringer lactate—a fluid that simulates bodily fluids and thus, the conditions encountered in the human body—and comparing the alloy’s corrosion behavior in that medium to that in a 3.5% NaCl aqueous solution (mass fraction). Secondly, to analyze the combined effect of grain refinement, crystallographic texture, and second-phase redistribution on the corrosion behavior and mechanical properties of ZK30 Mg alloys. This analysis was conducted to provide more exhaustive knowledge of the electrochemical properties of the UFG Mg alloy and to exploit its possible biomedical applications. The ZK30 Mg alloy had up to four passes of processing via ECAP, followed by an exhaustive analysis of microstructural evolution and crystallographic texture using scanning electron microscopy (SEM) equipped with EBSD technique to understand how ECAP has influenced the previous. Electrochemical measurements, such as open circuit potential (OCP), potentiodynamic polarization, and electrochemical impedance spectroscopy (EIS), were also conducted. The as-received material was annealed and tested as a reference.

## 2. Materials and Methods

**Figure 1:**
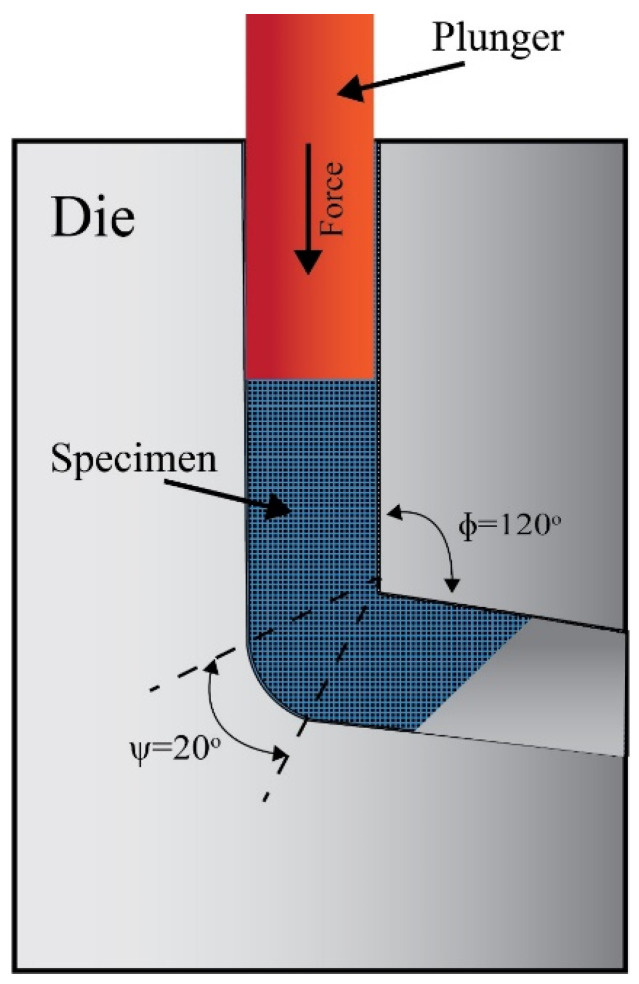
The schematic of the ECAP die.

Commercial ZK30 (Mg-3Zn-0.6 Zr-0.4 Mn, wt%) alloys were the material of choice. The samples were sectioned into 60 mm long billets with a diameter of 20 mm. Before processing, the samples were annealed for 16 h at a temperature of 430 °C to dissolve the second phase particles. The samples were processed via a die characterized by a vertical cylindrical channel intersecting an inclined one with an internal angle of intersection of 120° and with an outer arc of curvature of 20°, as shown in Figure 1. Pressing forces were applied to the samples using a ram with a speed of 10 mm/min. In that die, the billets were hot processed at a maintained temperature of 250 °C for either 1 pass (1-P), 2 passes of route Bc (2-Bc), or 4 passes of route Bc (4-Bc).

To accurately characterize the microstructural evolution, both the AA samples and the ECAP-processed samples had to be prepared. First, the billets were longitudinally sectioned. Second, the specimen was cold mounted in conductive epoxy. Third, the samples were grinded using 600, 800, 1000, and 1200 grit silicon carbide sandpaper. The samples were rinsed with water before each increment in grit number. Fourth, the samples were polished to a mirror-like finish. Initial polishing was done using 1 μm and 3 μm diamond suspensions and yellow DP-lubricant. Between polishing steps, the samples were ultrasonically cleaned in ethanol for 10 min and then blow dried completely. The final polishing stage was conducted using a colloidal silica suspension of particle size 0.05 μm. The samples were then put under an optical microscope and the last polishing step was repeated until the optical microscope showed a scratch-free surface. Both grinding and polishing were conducted on wheels spinning at 150 rpm. Fifth, the samples were then etched in an acidic solution of 6 g picric acid, 5 mL acetic acid (95%), 10 mL water, and 100 mL ethanol for 50 s. Finally, to remove etching stains or oxide layers from the surface of the samples, they were ion milled. To ion mill the samples, a 2 keV ion beam was used on a flat ion milling system spinning with a rotational speed of 0.425 s^−1^ at a specimen tilt angle of 85° (i.e., the sample’s surface and the ion beam axis had an angle of 5° between them) for 30 min.

This research investigation relied on a Scanning Electron Microscope equipped with an EBSD accessory for the characterization of the samples’ microstructure, as well as their crystallographic texture before and after processing. Elemental analysis of the samples’ compositions was conducted using Energy-Dispersive X-ray Spectroscopy (EDS) and X-ray fluorescence (XRF). EBSD samples were sectioned alongside the central longitudinal plane, parallel to the pressing direction. The axes of the reference system coincide with the extrusion direction (ED). EBSD measurements were taken from the top ED surface with a SU-70 SEM. The SEM operated at 15 kV and with a typical current of 1.5 nA. Crystallographic data was captured with the HKL Channel5 Flamenco software (Concord, MA, USA), which was also used to post process the data to generate the inverse pole figure (IPF) map. Data was captured with a step size of 100 nm. Finally, X-ray diffraction analysis (XRD) was used to determine the crystallographic structure of the samples. A JEOL JDX-8030 X-ray diffractometer (Joel Ltd., Tokyo, Japan) was operated at 40 Kv and 30 mA to perform XRD. The diffractometer used Cu-Kα radiation, and had a scan rate of 2 degrees/min.

Corrosion testing was conducted with a 3-electrode flat corrosion cell; the procedures and data recording were performed by an SP-200 Potentiostat (Bio-Logic-Lambda System Kreft Barszczewski Sp.J., Warszawa, Poland). The cell consisted of a counter electrode, which was a platinum mesh; a reference electrode, which was a saturated calomel electrode (SCE); and the working electrode, which was either the AA or the ECAP-processed ZK30 alloy samples. Firstly, the samples were sectioned into rectangular 20 × 30 mm pieces. Then, the samples were grinded and polished with 800, 1200, and 4000 grit silicon carbide sandpaper. Finally, the samples were cleaned with acetone and rinsed with deionized water to ensure the removal of any surface contamination, surface layers, or oils. Testing was conducted across two types of corrosive agents: ringer lactate and sodium chloride (3.5 mass% NaCl solution), both at room temperature. To minimize the ohmic drop and guarantee accurate results, this experiment made use of a Luggin capillary. The potential scan rate for the polarization technique was 0.166 mVs^−1^, thus guaranteeing a steady-state condition. Linear potentiodynamic polarization scans were applied with a potential window of ±250 mV against open circuit potential (OCP). Electrochemical impedance spectroscopy (EIS) was performed at OCP by applying a sinusoidal voltage between ±10 mV within a frequency range of 10 mHz to 100 kHz.

To assess the evolution of the alloy’s mechanical properties, Vicker’s microhardness tests (Hv) were performed. The tests were performed on the AA sample, as well as the 3 ECAP-processed ones. The test was conducted with an applied load of 0.5 kg, for 5 times over equispaced indentations each for 15 s, and then the hardness values were averaged for more precise results. The first indentation was at the billets’ outer edges, and testing moved towards the center with each indentation. Additionally, the samples had tensile tests conducted to assess their mechanical properties. Three specimens per processing path were tested at room temperature to ensure precise, descriptive results. The tensile samples were sectioned from the center of the billets, with appropriate geometry and dimensions based on the E8M/ASTM standard. The test was conducted using a 100 kN universal testing machine operating at a strain rate of 10^−3^ s^−1^.

## 3. Results and Discussion

### 3.1. Microstructure Evolution

**Figure 2:**
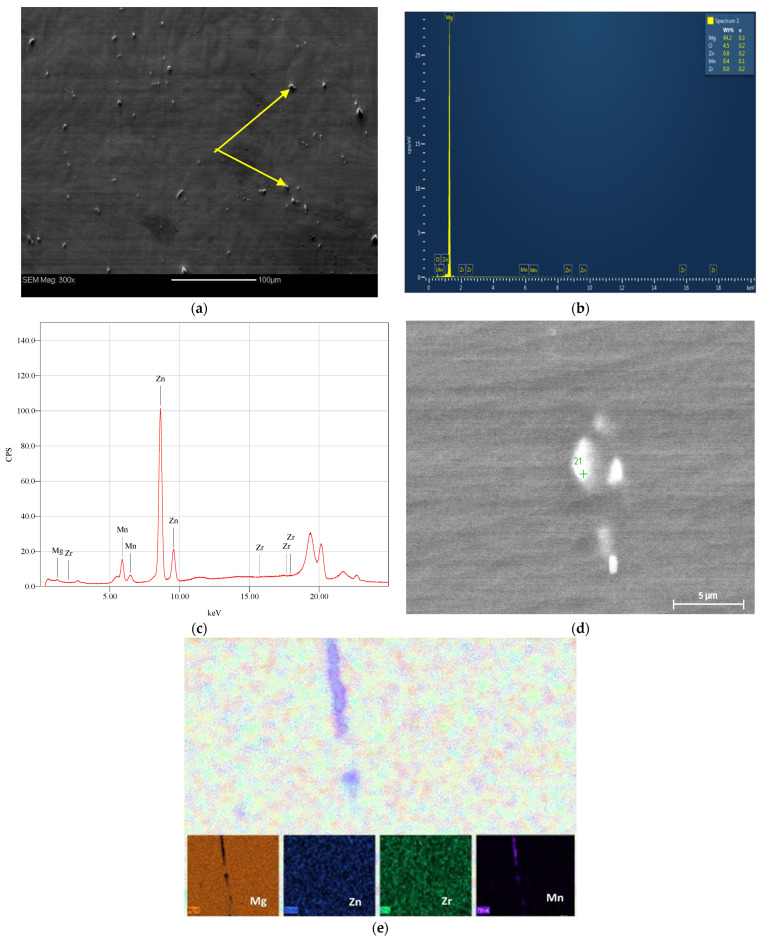
(**a**) SEM micrographs, (**b**) EDS analysis, (**c**) XRF analysis, (**d**) SEM for the EDS area, and (**e**) elemental mapping of the AA ZK30 alloy; arrows point at second phases.

**Figure 3:**
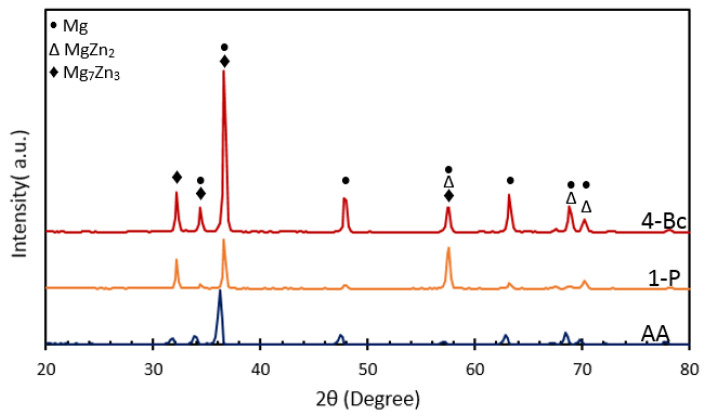
XRD pattern diffractions of the ZK30 alloy before and after ECAP processing through 1-P, and 4-Bc.

Figure 2 shows the SEM micrograph coupled with the EDS and XRF analyses of the AA billets of Mg alloy. The EDS and XRF analyses show the existence of Mg, Zn, Zr, and Mn elements in the alloy. Elemental mapping, depicted in Figure 2e, shows the distribution of Mg, Zn, Zr, and Mn. Figure 3 shows the XRD analysis which confirmed the presence of the α-Mg phase and indicated the existence of other second phases, namely, MgZn_2_ and Mg_7_Zn_3_ [5,13]. The α-Mg phase grains, as well as the secondary phases particles, are highlighted in Figure 2a. Orlov et al. reported that Mg-Zn secondary phases tend to agglomerate at grain boundaries (GBs) and at the triple junctions of matrix grains while the Zn-Zr phase grains tend to be distributed along GBs [13]. As depicted in Figure 3, the AA condition almost solely consisted of a single phase α-Mg solid solution. Only miniscule peaks of second phase precipitates were detected which can be attributed to the annealing process. Notably, ECAPing the samples using 1-P prompted the formation of second phases precipitates. The peaks corresponding to the second phases increased in number and intensity with the number of processing passes, up to 4-Bc. This increase is attributed to the recrystallization driven by SPD during ECAP [58]. The aforementioned findings agree with previous studies [19,58,59,60,61].

**Figure 4:**
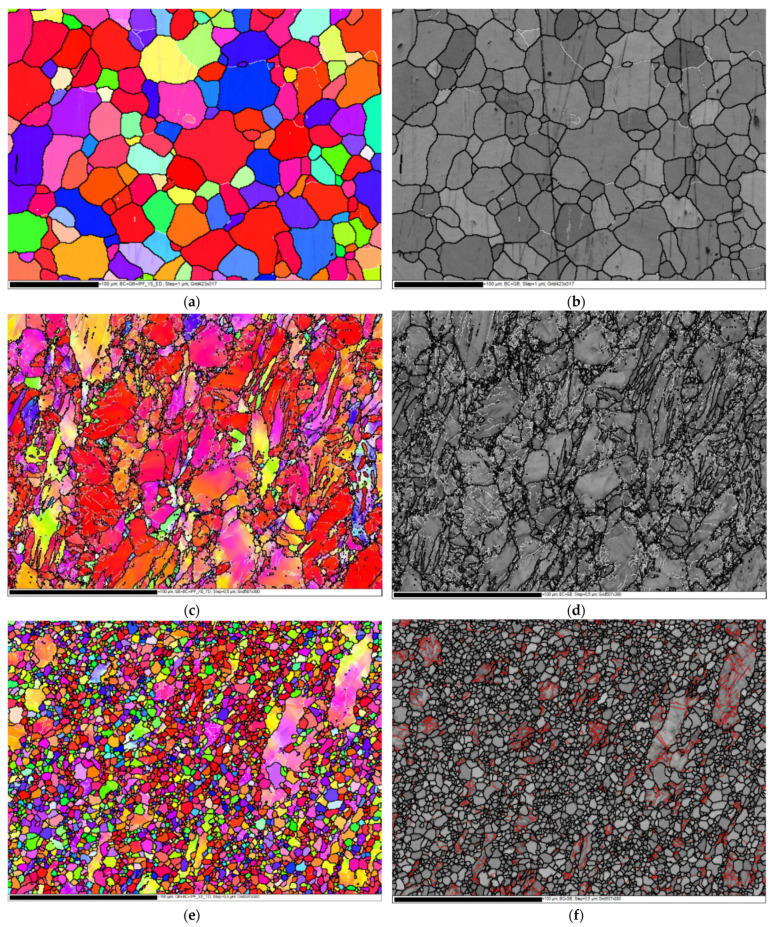

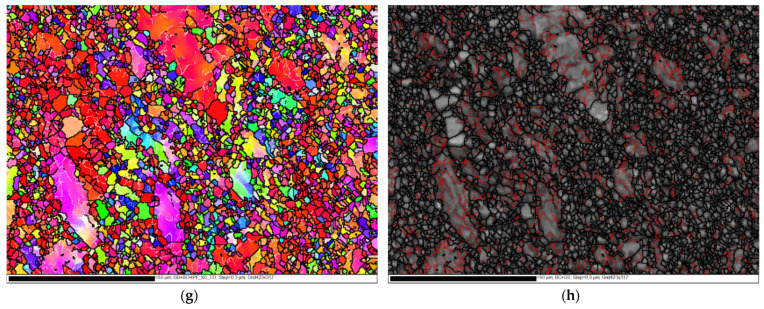
IPF coloring maps relative to ND and their corresponding BC maps with HAGBs in black lines and LAGBs in white lines (AA and 1-P) and red lines (2-Bc and 4-Bc), superimposed for the AA ZK30 billets (**a**,**b**), and ECAPed billets through (**c**,**d**) 1-P, (**e**,**f**) 2-Bc, and (**g**,**h**) 4-Bc.

**Figure 5:**
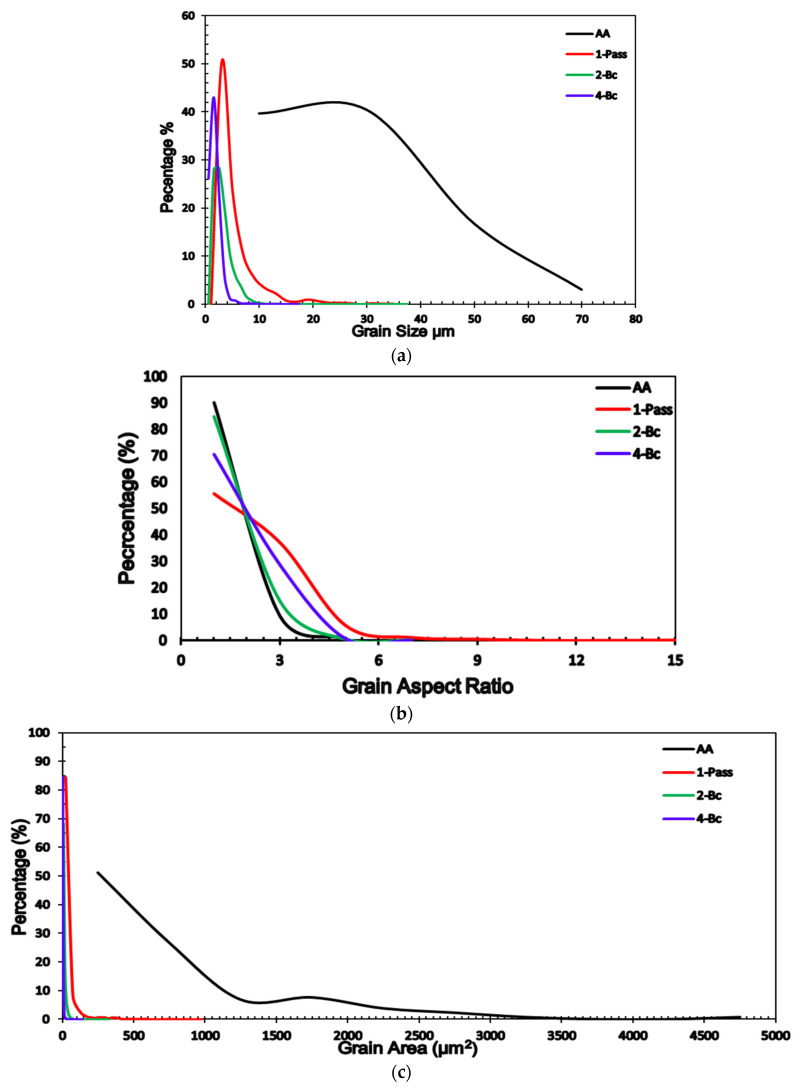
Relative frequency of (**a**) grain size, (**b**) grain aspect ratio, and (**c**) grain area distribution of the ZK30 alloy AA and ECAPed billets.

**Figure 6:**
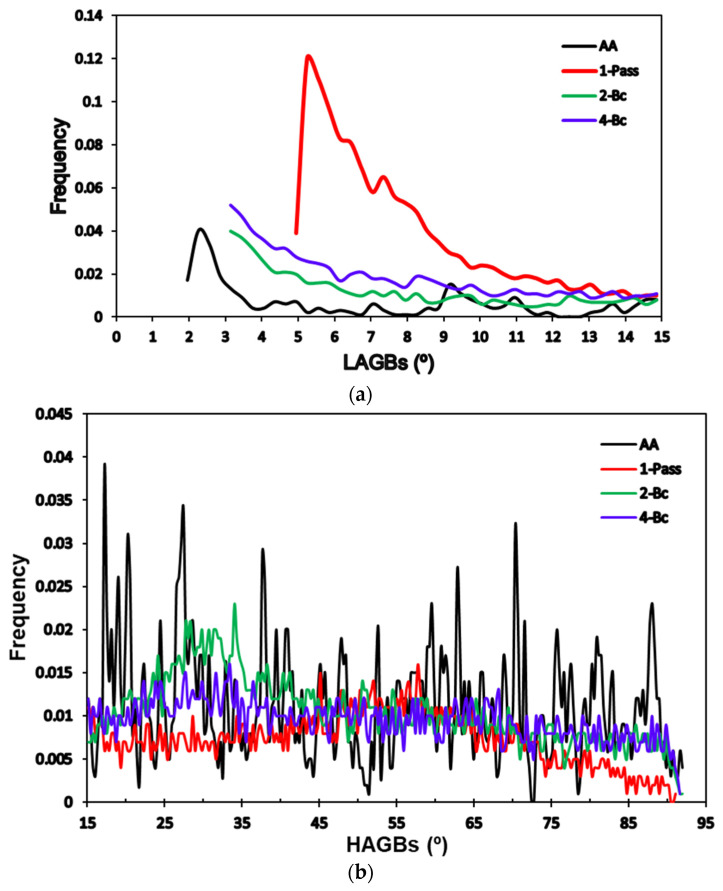
Relative frequency of the (**a**) LAGBs and (**b**) HAGBs of the ZK30 alloy AA and ECAPed billets.

**Figure 7:**
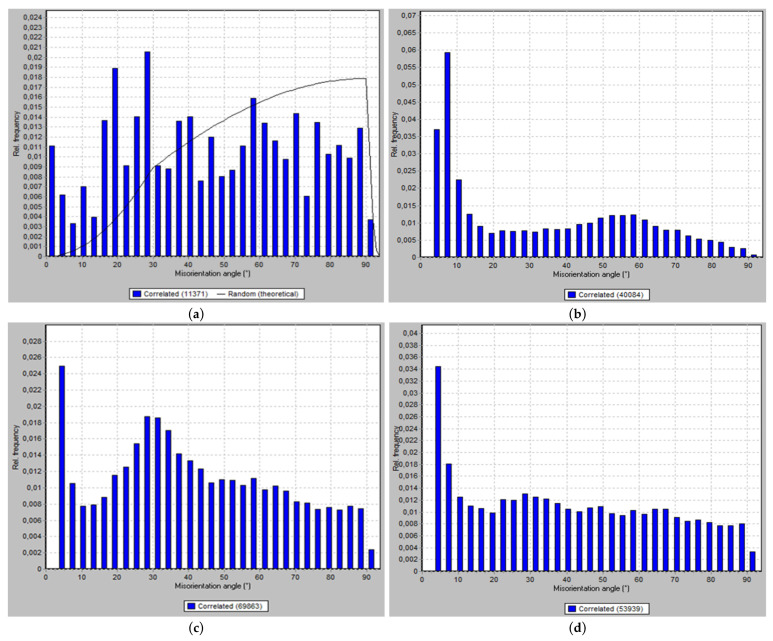
Misorentation angle distribution histograms obtained from the EBSD data for (**a**) AA and ECAPed billets processed through (**b**) 1-P, (**c**) 2-Bc, and (**d**) 4-Bc.

EBSD analysis was relied on in the investigation of the microstructural evolution and crystallographic texture of the ZK30 billets before (AA condition) and after ECAP processing using the previously mentioned processing conditions. Three different processing paths were investigated: 1-P, 2-Bc, and 4-Bc. Figure 4 contains the inverse pole figure (IPF) coloring maps and their corresponding band contrast (BC) maps for the four sample types. High-angle grain boundaries (HAGBs) were considered as those with an angle of misorientation larger than 15°, while low-angle grain boundaries (LAGBs) were considered to range between 3° and 15°, all relative to the TD (transverse direction). HAGBs are colored black in all four processing paths’ maps. For the AA and 1-P processing paths, LAGBs are depicted as white lines, while those of the other two are colored red. Table 1 shows the grain size, aspect ratio, and grain area data of the four types of samples. A comparison between the grain sizes, grain aspect ratios, and grain area distributions of the four samples is made in Figure 5. The LAGBs and HAGBs distribution in the four ZK30 alloy samples is depicted in Figure 6 and their misorientation histograms are shown in Figure 7.

The AA microstructure was dominated by equiaxed coarse recrystallized grains almost free of substructure grains with a low number of LAGBs, which is apparent in the GB map; this implies that the microstructure had fully recrystallized. On the other hand, some extremely fine grains, with sizes less than 5 µm, were observed between the coarse grains (Figure 4a,b). The AA grain sizes ranged from 3.39 µm to 76.73 µm with an average grain size of 26.69 µm. In addition, analysis of the AA billet’s grain sizes revealed an average grain aspect ratio and an average grain area of 0.46 and 729 µm^2^, respectively. From the IPF map, the AA sample can be seen to consist mostly of grains oriented in the 001/red orientation.

The 1-P processed sample had a completely different microstructure, as a result of the severe plastic deformation imparted by ECAP. The grains were severely elongated and oriented in the extrusion direction; however, the dominating grain orientation remained the 001/red orientation, as shown in Figure 4c. Although the structure was dominated by the elongated grains, very fine grains also existed, which is evidence of partial recrystallization. Thus, it can be concluded that processing resulted in partial dynamic recrystallization at the regions with high density of high grain boundaries (HAGBs > 15°) and so parts of the microstructure were divided into extremely fine grains. In addition, the density of LAGBs significantly increased by 267.7% compared with the AA sample, which can be observed in the GB maps (Figure 4d). As a result of processing via 1-P, the grains were refined. The grain size ranged from 2.24 µm to 35.22 µm with an average grain size of 5.43 µm, seen in Figure 5a and in Table 1. It is worth mentioning that the reduction in the average grain size after ECAP processing through 1-P correlates with the increase in the amount of LAGBs (LAGBs < 15°) (Figure 6a and Figure 7b). Lower random distributions for the HAGBs could also be observed as shown in Figure 6b. Dumitru et al. [61] reported similar behaviour for the ZK60 alloy. The average grain’s aspect ratio increased up to 2.09 which is an indicator that the grains had significantly elongated from the 1-P path processing (Figure 5b, Table 1). The average grain area was reduced to 37.14 µm^2^, as shown in Figure 5c, which proves the grain refinement accompanying the 1-P condition.

Further increase in the plastic strain up to 2-Bc showed significant grain refinement compared with the 1-P condition due to the accumulation of shear strain, as shown in Figure 4e and Figure 5a, where it can be observed that areas of coarse grains were significantly reduced. ECAP processing via 2-Bc revealed that grain sizes ranged from 1.13 µm to 37.33 µm with an average grain size of 3.17 µm (Table 1). Furthermore, the average grain aspect ratio was reduced to 1.55 and average grain area was reduced to 10.8 µm2 (Figure 5b,c). The significant grain refinement indicated that the imposed strain during 2-Bc is fairly enough to activate the dynamic recrystallization (DRX) process in the great majority of the areas and to form UFG grains. However, in some other areas it is required to increase the plastic strain to do so (Figure 4e). It is worth mentioning here that processing via 2-Bc was accompanied with a reduction in the LAGBs density by 60.6% compared with its 1-P counterpart, as shown in Figure 6a and Figure 7c, which can be attributed to dynamic recovery.

**Figure 8:**
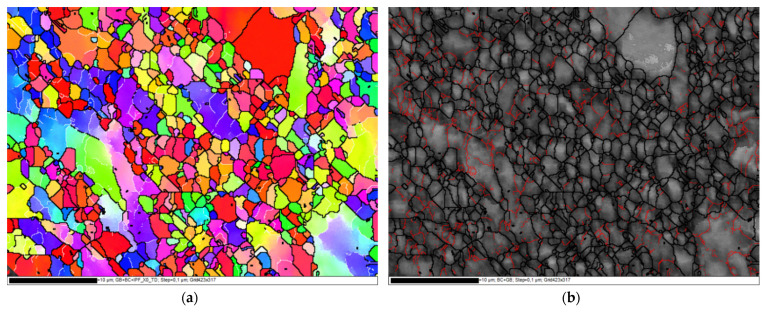
IPF coloring maps relative to ND and their corresponding BC map with high angle boundaries >15° in black lines and low angle boundaries 3–15° in red lines for the ZK30 billet processed through 4-Bc at higher magnification. (**a**) IPF coloring map (**b**) BC map.

Accumulation of the plastic strain in 4-Bc caused further refinement in the ZK30 billets (Figure 4g). Figure 8 shows the IPF maps relative to the TD and their corresponding BC maps at higher magnification. A geometric grain subdivision took place due to the SPD and was accompanied by grain refinement and a very high density of substructures, as clearly shown in Figure 8a. The grain sizes ranged from 0.76 µm to 17.86 µm with an average grain size of 1.92 µm (Figure 5a) which meant that 4-Bc processing experienced a reduction in the grain size by 92.8% compared with the AA counterpart. The significant refinement occurred by ECAP processing can be attributed to the continuing lattice rotations at GBs due to the shearing near them, owing to the lack of the sufficient slip systems required for homogeneous plasticity [62]. Furthermore, as shown in Figure 5b,c, a significant reduction in the average grain area was attained after 4-Bc as it was reduced by 99.55% compared with the AA counterpart, whereas the average grain aspect ratio was reduced to 1.69 (Table 1). From Figure 4h and Figure 6a, it can be observed that samples processed using the 4-Bc route experienced an increase in LAGBs by 109.5% compared with its 2-Bc counterpart, which can be attributed to the accumulation of the plastic strain. On the other hand, 4-Bc experienced an increase of 84% in HAGBs compared with the 1-P counterpart, which occurred as a result of the GBs transitioning from LAGBs into HAGBs. Dumitru et al. [61] reported that the accumulation of shear strain via increasing the number of ECAP passes leads to the accumulation and recombination of the dislocations which leads to the formation of subgrains and integrated fine equiaxed grains.

From the aforementioned findings, it was revealed that the density of LAGBs showed a significant increase after the first pass and then decreased in the subsequent passes which agrees with previous studies [5,61]. During ECAP processing, an enormous number of dislocations was created, they entangled with each other during ECAP, and then rearranged themselves to form LAGBs, thus increasing the total number of LAGBs [63]. During subsequent passes, the recrystallization process occurred and the LAGBs transformed into HAGBs, which caused a more stable structure to be formed. The bigger fraction of HAGBs in the 4-Bc condition compared with the 1-P counterpart clearly proves the previous. It is worth mentioning here that the transformation of LAGBs into HAGBs indicates the completion of the DRX process, [64] which strengthens the alloy as the HAGBs hinder the dislocations’ movement and block them [65]. These findings agree with an earlier study [61]. Figueiredo et al. [66] introduced a comprehensive model for microstructural evolution during ECAP processing of Mg alloys. They demonstrated that the microstructure of the Mg alloys evolves in the first pass into a bi-modal or multi-modal grain distribution and further processing through subsequent passes results in more refinement. Finally, a UFG homogeneous distribution microstructure should be attained after an adequate number of passes.

Similar findings were reported in earlier studies in terms of microstructural evolution of Mg alloys. Dumitru et al. [61] investigated the effect of ECAP processing with a die channel angle of 90° on the microstructural evolution and mechanical properties of ZK30 alloys. They reported that the microstructure remained heterogeneous having a bi-modal grain size distribution until the second pass; however, a fully equiaxed recrystallized microstructure was attained after the third pass of route Bc. Mostaed et al. [24] found that processing via route 4-Bc with a die channel angle of 110° at 250 °C caused remarkable grain refinement; however, some coarse grains were also attained. Processing via 4-Bc at 150 °C caused a completely recrystallized UFG microstructure with a 0.6 µm average grain size. They also reported that processing via 8-Bc at 150 °C resulted in grain growth and a UFG structure of 1 µm average grain size. Zheng et al. [64] had processed Mg-Zn-Ca alloys through up to 4 passes of routes A, Bc, and C (in route A, the ECAPed sample is processed repetitively without any rotation between the subsequent passes; and in route C, the sample is rotated 180° about its longitudinal axis between the subsequent passes [28]). They reported that route Bc was the most effective route in grain refinement and that it formed a homogeneous UFG structure with an average grain size of 0.7 µm. In term of misorientation angle, they found that the fraction of LAGBs decreased with increasing the number of ECAP passes and that route Bc experienced a lower fraction of LAGBs. Xie et al. [67] reported that AZ31 processed through 8 and 12 passes of route Bc exhibited grain sizes of 2.13 µm and 2.08 µm, respectively. However, the 12-Bc showed more equiaxed grains as some large, elongated grains, with an average grain size of 16 µm, were observed in the 8-Bc condition. Suh et al. [68] processed the AZ31 Mg alloy through 2 passes of route A, B, and D (route D; rotating the billets 90° in the same direction between the subsequent passes) with a die channel angle of 110°, and they found that 1-P refined the alloy’s average grain size from 14 to 8.4 µm while subsequent passes did not cause any further refinement. Gopi et al. [69] found that AM90 processed through 4-passes of route Bc with a die channel angle of 110° revealed a homogeneous and refined structure compared with the as-cast condition. The misorientation angles’ fractions increased almost all over the whole of the misorientation range. Ma et al. [70] found that 2-passes of ECAP processing via route Bc resulted in refining the ZAT522 Mg alloy from a grain size of 2.25 µm to 1.4 µm. Further processing through 4-passes resulted in refinement up to 1.18 µm. Xu et al. [71] demonstrated that processing the AZ91 alloy through 4-passes of route Bc resulted in DRXed grains with an average grain size of 7.3 μm. The base alloy, however, revealed an average grain size of 38.2 µm while further processing up to 12 passes caused further refinement up to 6.9 µm. 

### 3.2. Crystallographic Texture

**Figure 9:**
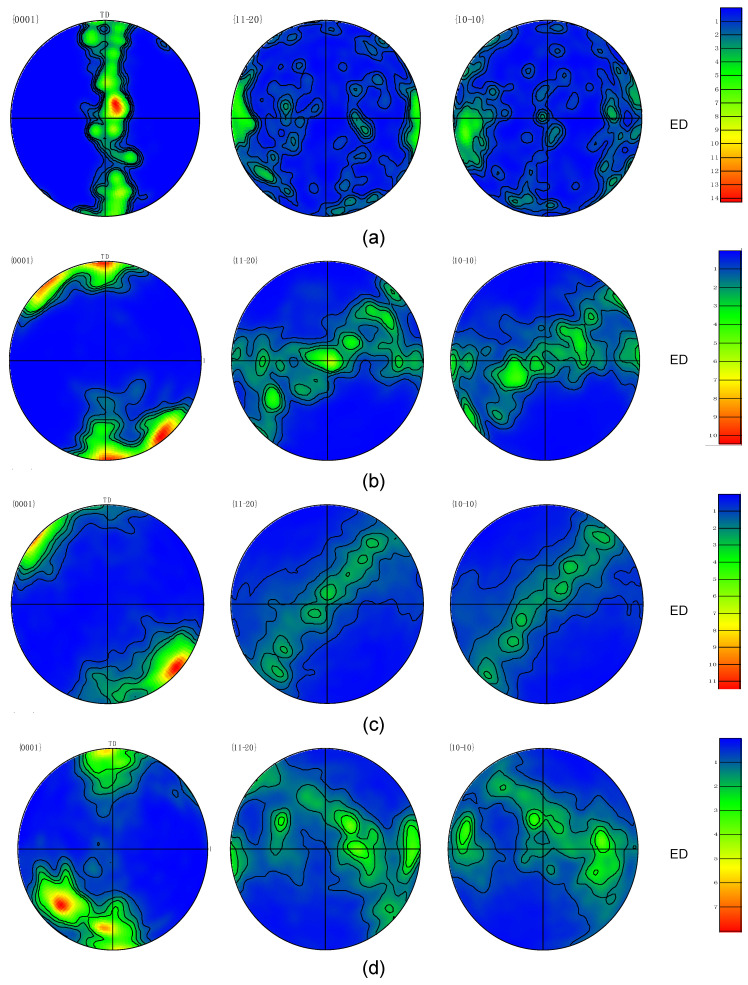
{0001}, {11-20}, and {10-10} pole figures showing the crystallographic texture of (**a**) AA, (**b**) 1-P, (**c**) 2-Bc, (**d**) 4-Bc.

Figure 9 contains the pole figures for the {0001}, {11-20}, and {10-10} planes of the ZK30 billets. In the AA condition, it was discovered that the majority of the {0001} basal planes were oriented parallel to the TD. The poles of the {11-20} and {10-10} were observed to be aligned parallel to the ED. ECAP processing altered the crystallographic texture of the ZK30 alloy and shifted the shear plane to 45°, relative to the ED. It is worth mentioning that the ideal simple shear texture in HCP metals corresponds to the active slip systems upon plastic deformation [26]. HCP crystal structures (Mg alloys) have three classes of slip systems: basal {0001} <11-20>, prismatic {10-10} <11-20>, or pyramidal {10-11} <11-20> slip [72,73].

After processing through 1-P, it was clear that the original fiber texture was replaced. Some {0001} basal planes were aligned parallel to ED and other {0001} planes rotated around the TD axis by almost 45°. This rotation means that the c-axes tilted 45° relative to ED and aligned with the shear plane normal (SPN); Figure 9b shows this rotation ({0001} pole figure). Two overlapping peaks can be distinguished on the {0001} pole figure (Figure 9b). Figure 9b also shows the {11-20} and {10-10} corresponding pole figures. The maximum texture intensity was reduced from 14 times random (AA billets) to a maximum texture intensity of 10 times random after ECAP processing through 1-P. The weakening in texture can be accredited to the limited number of slip systems during the 1-Pass upon shear deformation [71]. A similar texture was attained after 2-Bc processing and the {0001} basal planes tended to be tilted approximately 45° away from the ED because of the severe shear deformation imposed through route Bc (Figure 9c). This tendency can be attributed to the change of the shear planes’ position during processing passes. The 2-Bc path also showed a weak texture as the {0001} planes were aligned with ED. As revealed in Figure 9c, the maximum texture intensity had increased to 11 times random. The shift in the {0001} poles increased with the number of passes, as shown in the 4-Bc condition in Figure 9d. The maximum pole density of the {0001} was located 45° to the ED. A weak texture developed with the {0001} planes being aligned almost parallel to the TD.

Most notably, after 4-Bc it can revealed that the symmetry of the texture was almost lost, which was accompanied with a reduction in the texture intensity to 8 times random. The reduction in texture intensity in the ZK30 alloy with the increase in the number of ECAP passes is consistent with the texture evolutions of Mg alloys during ECAP [74] and pure Mg [75]. Finally, it is worth mentioning that the basal planes’ rotation was caused by the ECAP-induced shearing acting parallel to them [76]. Kim et al. have previously verified the rotation of most basal poles close to 45° from ED and TD by XRD [77]. These findings were in a good agreement with [76] for AM60 Mg alloy, [78] for the AZ31B Mg alloy, [79] for Mg-5.00Zn-0.92Y-0.16Zr alloy, and [64] for Mg-Zn-Ca.

### 3.3. Electrochemical Measurements

**Figure 10:**
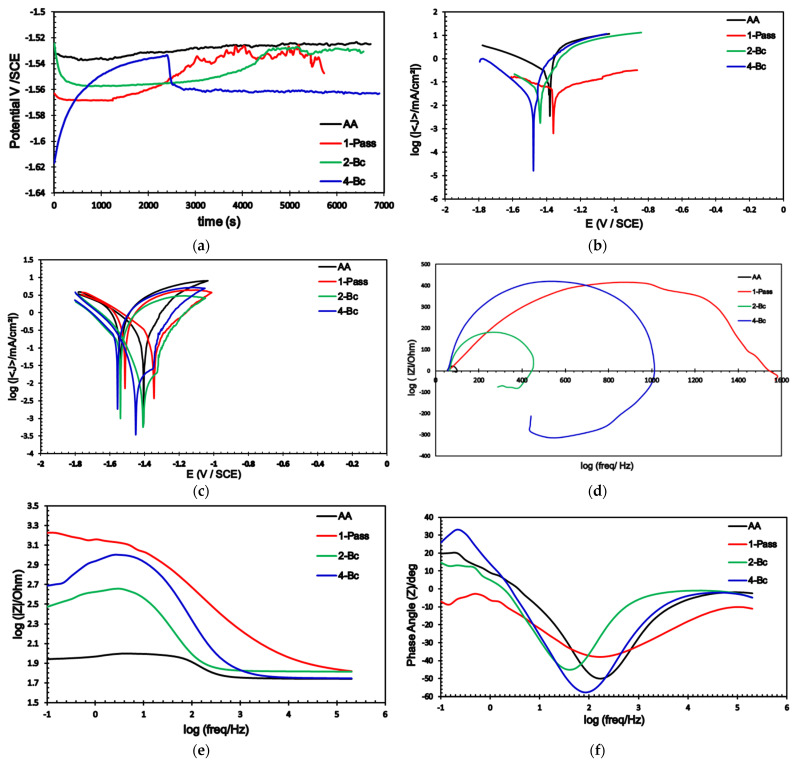
Corrosion measurements (**a**) OCP curves, (**b**) potentiodynamic polarization curves, (**c**) cyclic potentiodynamic polarization, (**d**) Nyquist plot, (**e**) Bode plot, and (**f**) Bode plots’ phase angle of ZK30 alloy using ringer lactate solution.

**Figure 11:**
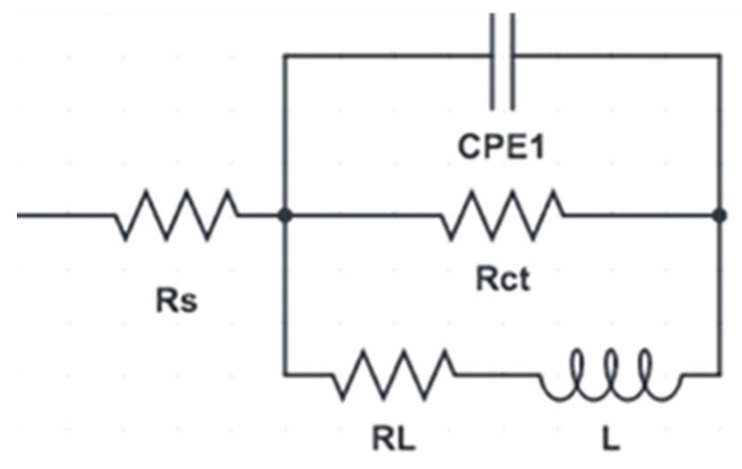
Equivalent circuit used to fit (EIS) data.

**Figure 12:**
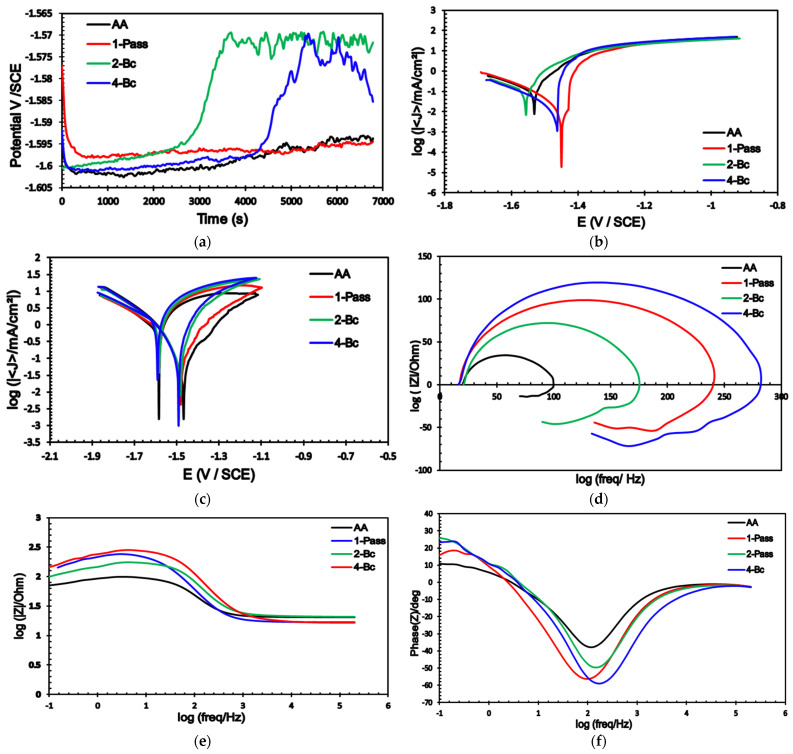
Corrosion measurements (**a**) OCP curves, (**b**) potentiodynamic polarization curves, (**c**) cyclic potentiodynamic polarization, (**d**) Nyquist plot, (**e**) Bode plot, and (**f**) Bode plot’s phase angle of ZK30 alloy using 3.5% NaCl solution.

Several electrochemical measurements were carried out on the ECAPed biodegradable ZK30 Mg alloy samples to explore the effects of ECAP processing on corrosion properties. The measurements were conducted on cells with two types of electrolytic solutions: a ringer lactate solution—which mimics human bodily fluids—with pH 6.5, and a 3.5% NaCl solution. Figure 10 shows the electrochemical response of the ZK30 billets in ringer lactate to OCP, potentiodynamic polarization, cyclic potentiodynamic polarization, and the EIS results: Nyquist plots, bode plots, and bode plots’ phase angle variation. The equivalent circuit used to fit (EIS) data is shown in Figure 11. Similar to Figure 10, which was obtained using a ringer lactate electrolyte, Figure 12 shows the electrochemical behavior of ZK30 alloy using 3.5% NaCl as a corrosive solution.

The OCP of the AA and ECAPed ZK30 was tested in cells with ringer lactate or NaCl solutions. The curves of both types of solutions are shown in Figure 10a and Figure 12a, respectively. In the ringer lactate solution, the AA billets’ corrosion potential stabilized at −1.53 V as shown in Figure 10a. 1-P processing resulted in a gradual increase in the potential and reached a relatively constant value of −1.536 V after 4000 s. Processing via 2-Bc resulted in a sample that took about 4400 s to reach a constant value of −1.58 V (Figure 10a). Finally, the 4-Bc processed sample’s corrosion potential increased gradually up to −1.536 V and then dropped significantly; the sample finally stabilized at a corrosion potential of −1.56 V. The tendency of AA ZKxx alloys to shift towards nobler corrosion potentials in simulated body fluids was reported earlier by Mostaed et al. [24]. From the OCP curves of the NaCl electrolyte, Figure 12a, the AA condition had an apparent decrease in the corrosion potential down to −1.6 V, followed by constant small increases in value until the potential stabilized at −1.595 V after 4500 s as shown in Figure 12a. Similar behavior was noted in the 1-P condition. However, the 2-Bc and 4-Bc conditions demonstrated similar behaviors to one another of having a drop in their potential, followed by a sharp increase in the potential up to −1.57 V after 3500 s and 5100 s for 2-Bc and 4-Bc, respectively (Figure 12a).

It is worth mentioning here that Yang et al. [30] reported the sudden decrease in the potential in the early stage of the OCP test and the gradual increase that followed in the subsequent stages for AZ91 Mg alloy. The decrease in the potential in the early stages of OCP testing can be attributed to the breakdown of the oxide layer formed by air, as well as the non-uniform distribution of gains. After sufficient time, another passive protective layer forms which leads the potential to increase and stabilize. Similar to the ringer lactate solution samples, the shift of the AA ZKxx alloys towards nobler corrosion potentials in the 3.5% NaCl solution was reported earlier by Li et al. [43]. However, the difference in potential behavior between the AA and ECAPed billets was trivial when using either the ringer lactate or the NaCl solution; thus, it was not significant enough to imply nobler behavior of the ZK30 alloy sample. However, the sodium electrolyte seems to be more corrosive, which is evident from potentiodynamic tests’ results. Having said that, further investigations are required to confirm and explain the corrosion behavior of the ZK30 alloy. 

The potentiodynamic polarization curves (Tafel plots) of the AA and ECAPed ZK30 tested using ringer lactate or NaCl solutions are shown in Figure 10b and Figure 12b, respectively. Moreover, the corrosion current density (Icorr), corrosion potential (Ecorr), Tafel’s anodic and cathodic constants (βa and βc), and the corrosion rate in mils penetration per year (mpy) are derived from the Tafel plots and presented in Table 2. It is worth mentioning here that Icorr is a dependable means of investigating the corrosion resistance [43].

**Figure 13:**
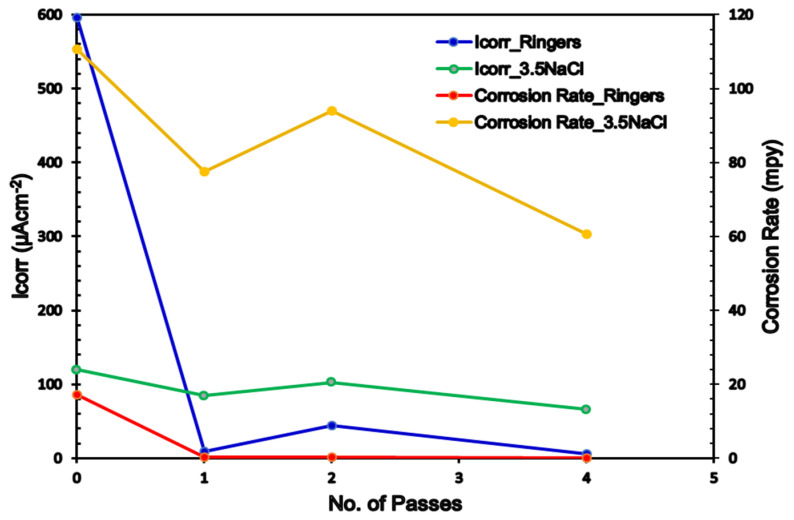
Comparison between the corrosion rate and Icorr for ZK30 in ringer and 3.5% NaCl solution.

As shown in Figure 10b and listed in Table 2, the 1-P condition sample tested using the ringer lactate solution had a significant Icorr reduction of 98.47%, compared with its AA counterpart; this was accompanied by a significant noble shift in the Icorr towards lower current densities. Further processing via 2-Bc yielded a slight increase in Icorr compared with its 1-P counterpart. Increasing processing up to 4-Bc resulted in a further reduction of Icorr of 99% compared with the AA condition (Figure 10b). The reduction in Icorr is a reliable indicator of the decrease of the corrosion rate. In addition, ECAP processing through 1-P caused a notable noble shift in Ecorr compared with the AA curve; whereas the 2-Bc and 4-Bc conditions caused an insignificant shift to higher negative values compared with their AA counterparts, as shown in Figure 10b. Furthermore, from Table 2, it can be observed that the 1-P sample experienced a significant reduction in the corrosion rate of 98.46% compared with the AA condition. Increasing the ECAP passes up to 2-Bc caused a slight decrease in the corrosion rate compared with the 1-P sample as the corrosion rate decreased only by 97.66%, compared with the AA condition. On the other hand, accumulating the strain up to 4-Bc resulted in a further reduction in the corrosion rate of 99% compared with its AA counterpart. The drop in corrosion rate after the first pass was reported by Peron et al. [50] for an AZ31 alloy tested using a simulated body fluid. Similar findings were achieved while using the 3.5% NaCl solution, as seen in Figure 12b. ECAP processing yielded reductions in Icorr of 29.85%, 14.7%, and 45.17% for the 1-P, 2-Bc, and 4-Bc samples, respectively, compared with the AA condition. Several investigations reported similar findings of the decrease in the Icorr with the number of ECAP passes [44]. In addition, Figure 13 shows a comparison between Icorr values and corrosion rates of ZK30 billets before and after ECAP processing in ringer lactate and 3.5% NaCl solutions. The 3.5% NaCl solution caused a higher corrosion rate compared with the ringer solution across all processing conditions. The AA billet tested using the NaCl solution had a significantly high corrosion rate increase of 540.9% of the AA billets tested using ringer lactate, as evident from Table 2. Similar findings were achieved for the 1-P, 2-Bc, and 4-Bc samples, as shown in Table 2 and sketched in Figure 12. This higher corrosion rate can be attributed to the increase in chloride ion concentration. On the other hand, 1-P processing (in NaCl solution) experienced a reduction in the corrosion rate of 29.9% compared with the AA counterpart, as shown in Figure 12b and displayed in Table 2. Similar to the 2-Bc billets which were tested in ringer lactate, increasing the number of processing passes to 2-Bc in NaCl resulted in an increase of 21.25% in the corrosion rate, compared with their 1-P counterpart. Further processing through 4-Bc revealed a reduction again in the corrosion rate by 45.25% compared with the AA counterpart.

Despite the improvement in corrosion resistance from grain refinement, it was reported elsewhere [50] that significant refinement of the grain from multiple passes of ECAP resulted in a deterioration in the corrosion resistance. This can be explained with the help of the texture evolution within the material as seen in Figure 9. The enhanced corrosion resistance after ECAP processing for both the ringer lactate and NaCl samples indicates that the fine-grain billets were noble while the coarse-grained ZK30 billets were active. The uniform distribution of the second phases after ECAP processing through multiple passes played an important role in the corrosion inhibition of Mg alloys, as reported by Alateyah et al. [19]. Cubides et al. [44] explained the improvement in the corrosion resistance of Mg–9Al–1Zn alloys due to the grain refinement resulting from ECAP processing as well as the formation of a coherent and protective oxide layer. Li et al. [43] found that ECAP processing for ZK60 alloys mitigated any signs of localized corrosion because ECAPing results in a homogenous distribution of anodic and cathodic sites on the fine-grained alloy, and thus prevents the formation of a potential gradient across the surface of the sample. 

In addition, cyclic potentiodynamic polarization (CPD) was also carried out to study the effect of ECAP processing on the ZK30 alloy’s passivity capabilities, and to predict the potential of localized corrosion occurrence. As shown in Figure 10c and Figure 12c, it is clear that all ZK30 samples completed the hysteresis loop, which indicates the occurrence of re-passivation and shows the protection potential [80]. In the CPD curves, the forward scan of the potential shows regions of active corrosion and passivation and the backward (reverse) potential scan shows regions of pitting and re-passivation [81]. 

From Figure 10c and Figure 12c, the Ecorr in the forward scan was nobler than the reverse scan in all processing conditions. The previous indicates that ZK30 billets are very susceptible to re-passivation and that the passive layer formed will be resistant, which agrees with [82]. In addition, the 1-P processed sample with ringer lactate showed high re-passivation potential (Erep), as shown in Figure 10c, indicating more resistance to pitting corrosion. On the other hand, the values of Erep for the ZK30 samples tested using 3.5% NaCL solution were extremely similar to each other, as shown in Figure 12c. After the forward scan, approximately constant current densities (passive region) were found in Figure 10c and Figure 12c. As shown in Figure 10c, the passive region extended from −1.04 to −1.44 V for ringer lactate solution while it extended from −1.14 to −1.54 V, as shown in Figure 12c. Similar findings were attained in [19] for the AZ31 Mg alloy. The formation of the passive region can be attributed to the formation of a protective oxide layer on the surface of ZK30 billets. The extent of this passive layer was constant and not a function of the sample’s grain size. In addition, ECAP processing reduced the Icorr in the passive region for both ringer lactate and NaCl solution which indicates that more protective layers formed on the surface of ECAPed billets, which agrees with [83]. Song et al. reported that refining the grains of Mg alloys resulted in improving the re-passivation capacity [84]. 

The corrosion resistance of the ZK30 alloy was assessed using EIS analysis to support the potentiodynamic polarization findings and to prove the increase in the protective ability of the oxide layer with the increase in the imposed strain. The EIS data were fitted using the equivalent circuit shown in Figure 11. The Rs and Rct, are the solution and charge resistances. The CPE corresponds to the double-layer capacitance, and RL and L are the pitting resistance and inductive response. Nyquist plots of the AA as well as the ECAPed ZK30 billets are shown in Figure 10d for the samples submerged in the ringer lactate solution and in Figure 12d for the samples with the 3.5% NaCl solution. As shown in Figure 10d and Figure 12d, all the ZK30 billets displayed capacitive semicircles which can be attributed to both the ZK30 billets’ resistance to charge transfer and the double-layer capacitance, as stated in [83]. As seen in Figure 10d and Figure 12d, it is clear that the AA billets response was a very small semicircle compared with that of the ECAPed billets across all corrosive solutions. The previous indicates improvement in corrosion resistance after ECAP processing due to the obtained UFG after processing which agrees with the potentiodynamic polarization findings. For the ringer lactate corrosive solution, ECAP processing through 1-P revealed a significant increase in the semicircle diameter (Figure 10d). On the other hand, increasing the processing passes up to 2-Bc resulted in decreasing the semicircle diameter compared with 1-P, as shown in Figure 10d. It is worth mentioning here that increasing the ECAP passes up to 2-Bc resulted in a significant increase in the dislocation density which led to a decrease in the corrosion resistance; this agrees with earlier studies [5,85,86]. Furthermore, the accumulation of strain from processing to 4-Bc caused a significant increase in the semicircle diameter, compared with its 2-Bc counterpart. 

Similar findings were recorded for the 3.5% NaCl solution, as shown in Figure 12d. Processing through 1-P displayed a very large capacitive arc compared with its AA counterpart. This increase in the semicircle diameter with 1-P was followed by a notable decrease after 2-Bc, as shown in Figure 12d. 4-Bc processing experienced a significant increase in the capacitive arc even compared with its 1-P counterpart. As the previous literature indicates, ECAP processing results in the formation of a stronger oxide film; increasing the ECAP passes also results in an increased oxide film thickness, and both lead to enhanced corrosion resistance [5,19,44]. To analyze and explain the electrochemical response of the ZK30 alloy, the EIS spectra of ZK30 billets were fitted to an electrical circuit (Figure 11). The electrical parameters of the EIS equivalent circuit for ringer lactate and 3.5% NaCl solutions are shown in Table 3. The solution resistance is denoted by Rs, the constant phase corrosion element (CPE1) describes the capacitance of the oxide film, the double layer charge transfer resistance at the ZK30 alloy/solution interface is denoted by Rct, the pitting resistance is denoted by RL, and the inductance is denoted by L. As shown in Table 3, it can be concluded that ECAP processing increased the pitting resistance for all the processed billets compared with their AA counterparts. For the ZK30 sample tested using ringer lactate solution, processing through 1-P caused a significant increase of 3414.4% in the pitting resistance, compared with the AA condition. Processing via 2-Bc reduced the pitting resistance by 68.6% compared with its 1-P condition counterpart; this matches the findings extracted from Figure 10d. On the other hand, the 4-Bc processed sample experienced a significant increase of 95.25% in the pitting resistance compared with the 2-Bc condition. Similar findings were noted for the NaCl solution. A huge enhancement in the pitting corrosion of 176.76% was noticed after 1-P, compared with the AA counterpart. Processing through 2-Bc and 4-Bc revealed increases of 86.77% and 114.76% in the pitting corrosion, respectively, compared with their AA counterparts. By comparing the EIS findings of the two corrosive solutions, the pitting corrosion of ZK30 improved more in ringer lactate. Pitting corrosion values in ringer lactate showed enhancements of 471.66%, 166.3%, and 352% during processing via 1-P, 2-Bc, and 4-Bc, respectively, compared with the NaCl findings. Generally speaking, the 1-P condition revealed the best corrosion resistance in both ringer lactate and 3.5% NaCl solutions. It can be concluded that the significant refining of the grain size during the first pass (as shown in Table 1) played a vital role in enhancing the corrosion resistance and corrosion rate across all corrosive solutions, as shown in Figure 10b,d and Figure 12b,d. Increasing the processing strain up to 2-Bc increased the dislocation density which affected both corrosion resistance and corrosion rate. Further processing through 4-Bc improved the corrosion resistance and corrosion rate compared with 2-Bc, which can be attributed to the UFG structure that resulted from 4-Bc (Table 1) which may enhance the thickness and coherency of the shielding passive oxide layer [13,84]. Accordingly, improvements in the oxide layer lead to better protection against pitting corrosion [19]. On the other hand, the decrease of the corrosion resistance after further passes, compared with the first pass value, can be attributed to the increase in dislocation densities and the dislocation buildup which stores abundant internal energy as grain boundary energy [86,87].

Furthermore, Figure 10e shows the Bode plots of ZK30 using ringer lactate as a corrosive solution. From Figure 10e, it is clear that all ECAPed billets show higher corrosion resistances than AA billets. In addition, the figure revealed that the 1-P condition displayed the highest corrosion resistance at all frequencies, while the 4-Bc condition had higher corrosion resistance compared with 2-Bc at lower and even intermediate frequencies up to 3 Hz, after which 2-Bc showed higher impedance. Similar findings were reported by Gu et al. [88]. At lower frequencies (of up to 0.4 Hz), the 4-Bc sample had higher phase angles. At intermediate frequencies (from 0.4 up to1.7 Hz), the AA billets possessed the highest phase angle. Above 1.7 Hz, 2-Bc showed the highest phase angle as shown in Figure 10f. On the other hand, for samples tested in NaCl solution, the 4-Bc billets experienced the highest impedance at lower and intermediate frequencies (up to 3 Hz), while the 2-Bc condition possessed the highest impedance at high frequencies (above 3 Hz), as shown in Figure 12f. Considering the phase angle, 2-Bc had the highest phase angle at low frequencies (up to 1 Hz) while AA billets had the highest phase angle at higher frequencies (above 1 Hz), as shown in Figure 12f. Accordingly, the impedance of the ECAPed billets, highlighted in Figure 10e and Figure 12e, confirmed that ECAP processing resulted in enhanced corrosion resistance.

**Figure 14:**
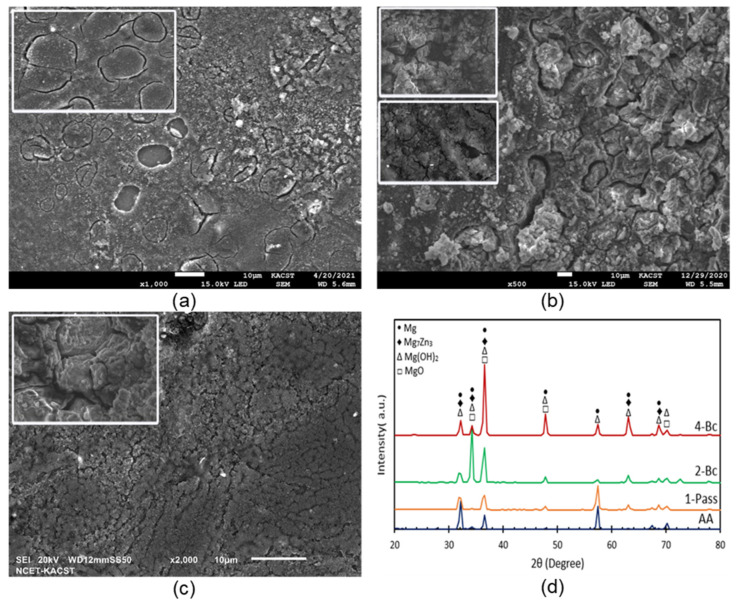
(**a**–**c**) SEM micrographs of the ZK30 alloy after corrosion test for (**a**) AA, (**b**) 1-P, (**c**) 4-Bc and (**d**) X-ray diffraction patterns of ZK30 alloy after corrosion.

Processing through ECAP via multiple passes resulted in the generation and multiplication of dislocations. These dislocations play a vital role in improving the corrosion behavior of alloys to be more noble [88,89]. These defect sites promote the development of oxide and hydroxide protective layers, such as MgO and Mg(OH)_2_ [5,19,22,90]. Peron et al. [50] attributed the increase in corrosion resistance after ECAP processing to the increased stability of the oxide layer associated with fine grains. Increasing the SPD through increasing the number of passes increases the thickness of the protective layer, as reported in [5,19,90]. To verify the previous, SEM was used. Figure 14 shows low and high magnification SEM micrographs of the AA and ECAPed ZK30 Mg alloy billets after corrosion testing using ringer lactate solution. In addition, Figure 14d shows the XRD pattern of the AA and ECAPed billets of ZK30 alloy after corrosion testing. Notably, Figure 14d confirmed that more MgO formed after ECAP. Furthermore, ECAP processing through 1-P enhanced the protective layer. Increasing the imposed strain up to 4-Bc resulted in strengthening the passivation layer. As more MgO peaks appeared in 4-Bc, a denser and more coherent protective layer, compared with its AA counterpart, was formed, as shown in Figure 14c. This led to the improvement of the corrosion resistance of the alloy. It is worth mentioning here that the SEM findings are in good agreement with both Tafel’s plots and the EIS findings (Figure 10) and match earlier studies [22,89]. Furthermore, the XRD pattern confirmed the formation of the passivation protective layer of Mg(OH)_2_ and MgO, as shown in Figure 14d. Similar findings were reported in an earlier study [86]. In addition, the XRD pattern of the ZK30 alloy confirmed the presence of the second phase Mg_7_Zn_3_, which plays an important role in improving both the corrosion resistance and strength of the alloy because it behaved as a protective barrier. In addition, the intensity of the second phase peaks increased with increasing the number of ECAP passes which agrees with earlier studies [22,44]. Accordingly, the second phase strengthening mechanism had an effective role in enhancing the corrosion and mechanical properties of the ECAPed ZK30 alloy by forming high potential phases [19]. Similar findings were reported in earlier studies. Tang. et al. [91] attributed the dramatic increase in the peak intensity of β-Mg17Al12 phase to the effect of ECAP in promoting the precipitation of the second phase Mg17Al12 particles from the α-Mg matrix of AM80 alloy. Habbale et al. [45] reported that the lower pH level of 3.5% NaCl led to severe pitting corrosion and AZ80/91 displayed deep corrosion attacks over the surface of the sample compared with the samples tested in a higher pH level (2.5% NaCl). On the other hand, they reported that the corrosion inhibition was attributed to UFG structure and to the distributed secondary phases resulting from ECAP processing which caused a low corrosion rate. Cubides et al. [44] found that increasing the number of ECAP passes leads to more coherent protective oxide layers, which results in more resistance to the localized breakdown of the aggressive species by blocking active anodic and cathodic sites. Sadawy et al. [83] reported that the decrease in the grain size resulting from ECAP processing leads to a decrease in the amount of impurities segregated at the grain boundaries, which improves the corrosion properties of the alloy. Torabi et al. [54] reported the formation of a protective oxide layer on the surface of Mg-HA bionanocomposites which improved the corrosion resistance of the composite.

### 3.4. Mechanical Properties

**Figure 15:**
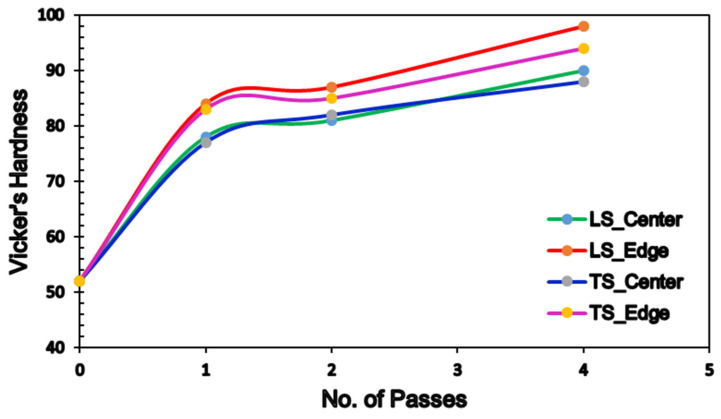
Distribution of VH across LS and TS of ZK30 billets as a function of number of passes.

Vicker’s hardness values (HV-values) were assessed along the longitudinal section (LS) as well as the transverse section (TS) of the samples; the values are plotted in Figure 15 and tabulated in Table 4. Figure 15 reveals that the HV showed almost matched distribution along both the LS and TS in both the peripheral regions (PR) and central regions (CR). The AA billets had seemingly identical hardness values across the LS and TS with an average value of 52 HV. Processing through 1-P revealed a significant increase in the HV-values by 38.5% and 59.6% in the CR and PR along the LS, respectively, compared with the AA condition, whereas the HV-values recorded 46% and 57.7% increase in the same regions compared with the AA counterpart. There is an additional increase of 12.5% and 3.6% in the CR and PR along the LS, respectively, when it is put in comparison with the 1-P condition. A similar trend was attained in the TS.

Accumulation of the strain through increasing the passes up to 4-Bc displayed additional increase in the HV-values in both the LS and TS, as shown in Figure 15. In the LS, it is clear that the HV-values increased by 73% and 86.5% in the CR and PR, respectively, compared with the AA counterpart in the LS, whereas the TS showed 67.3% and 78.8% increases in the HV-values in the same region, compared with the AA counterpart. Accordingly, the increase in the HV-value in the PR compared with CR can be attributed to friction between the ECAPed billets and the internal walls of the die [5,26]. In addition, the increase in the HV-values in the LS compared with the TS can be attributed to the intense texture resulting from the extrusion process, as the great majority of the basal planes were aligned parallel to the ED as reported by Mostaed et al. [24]. The increase in the HV-values of the ECAPed billets compared with the AA condition can be attributed to the grain refinement, as shown in Figure 4. Furthermore, increasing the imposed strain by increasing the ECAP passes resulted in an increase in the HV-values due to the UFG structure obtained after 4-Bc, as shown in Figure 4g; the previous indicates that the grain refinement strengthening mechanism was the dominant texture strengthening mechanism. It is worth mentioning here that further ECAP passes increase the dislocation density and thus impede dislocation motion [26]. Accordingly, strain hardening plays an important role in strengthening Mg alloys processed through ECAP, hence increasing the HV-values as reported in an earlier study [87].

**Figure 16:**
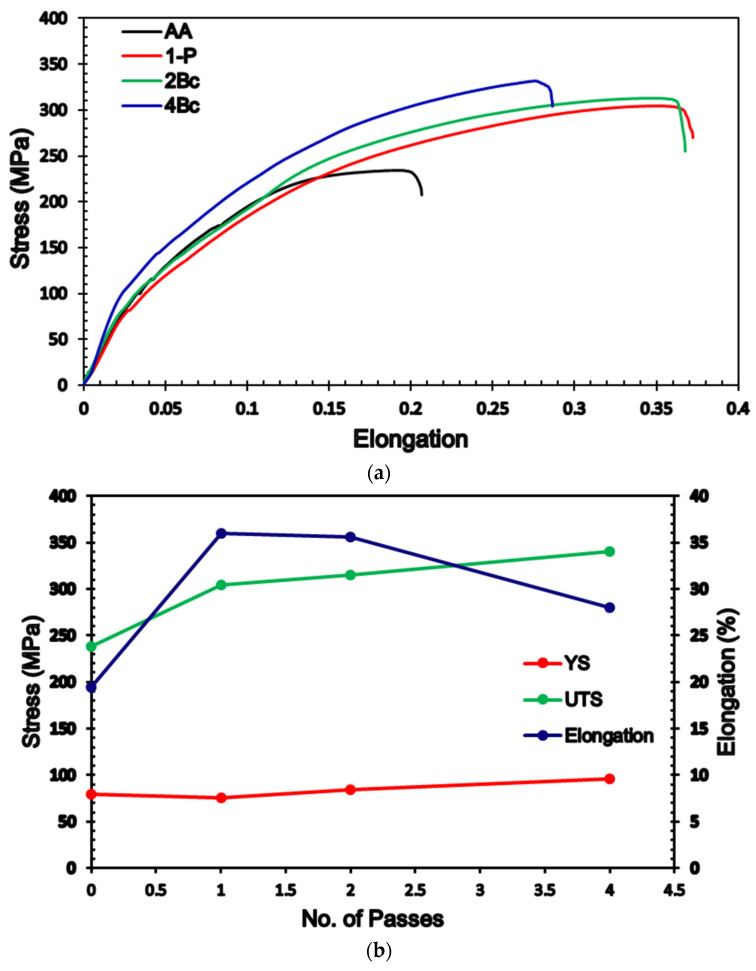
(**a**) Stress-strain curves and (**b**) variation of UTS, YS, and elongation % of ZK30 billets as a function of number of passes.

The stress-strain curves of the AA and ECAPed billets of ZK30 alloy were plotted as shown in Figure 16a. In addition, the tensile properties of the alloy as a function of increasing the processing passes were displayed in Figure 16b and tabulated in Table 4. From Figure 16 and Table 4, it was clear that ECAP processing resulted in an insignificant increase in the yield strength (YS) of 7.5% and 10% during processing through 1-P and 2-Bc, respectively, compared with the AA counterpart. On the other hand, 4-Bc revealed a 20% increase in the YS compared with the AA condition. The small increase in the YS after ECAP processing, despite achieving significant grain refinement (Figure 4g compared with Figure 4a), confirmed that the crystallographic texture had affected the YS of the ZK30 Mg alloy, which agrees with earlier studies [26,76]. Furthermore, the YS of the Mg alloy could also be affected by the activation of non-basal slip systems, as confirmed by Lie et al. [91]. On the other hand, ECAP processing experienced a notable increase in the ultimate tensile strength, as shown in Figure 16. 1-P processing resulted in increasing the UTS strength by 27.7% coupled with a significant increase of the elongation-to-failure (EL) by 81.3%, compared with the AA condition. Further processing through 2-Bc resulted in an additional increase in the UTS of 3.6% coupled with an insignificant decrease in the EL (Table 4). Strain accumulation up to 4-Bc experienced a notable increase in the UTS of 42.8% coupled with an increase of the EL by 37.2%, compared with the AA counterpart. On the other hand, it was clear that 4-Bc resulted in decreasing the EL by 24.3% compared with the 1-P counterpart.

Indeed, the increase of the UTS can be attributed to the significant reduction in grain size by increasing the number of passes, according to the Hall-Petch relationship, which indicated that the grain boundary mechanism dominated the texture strengthening mechanism. Accumulation of the imposed strain, via increasing the number of processing passes, leads to absorbing the LAGBs and it gradually transformed into more stable HAGBs, as shown in Figure 4, which resulted in the formation of finer grains [26]. Furthermore, grain refining leads to an increase in the GBs area which plays an important role in hindering the dislocation motion and increasing the UTS of the Mg billets, as reported by Cheng et al. [92]. The increase of the second phase particles, as well as the increase of the dislocation density by increasing the number of ECAP passes could have also resulted in increasing the UTS, which agrees with Dumitru et al. [61]. Furthermore, the slight increase in the UTS after the second pass can be attributed to the possibility of the occurrence of a dynamic recrystallization that led to dislocation annihilation. Static recrystallization between ECAP passes can also result in strain softening which leads to the slight increase in the UTS. Additionally, the improved ductility, displayed in Figure 16a and Table 4, can be explained by the bi-modal grain structure shown in Table 1. The fine grains (less than 5 µm) lead to an increase in strength; on the other hand, the large grains (around 20 µm) supported the deformation to large strains through providing strain hardening, which agrees with earlier studies [76,93,94]. Therefore, the special textures (Figure 9), along with the basal slip could improve the ductility of the ZK30 ECAPed billets, as shown in Figure 16 [76]. In addition, the improvement in the EL after ECAP processing can be explained by the increase of HAGBs which enhance grain boundary sliding [61]. The tensile findings were consistent with Dumitru et al. [61] when they processed ZK60 through up to 4-passes at 250 °C. They reported that 4 passes of ECAP resulted in improving the ductility by 30% coupled with a slight increase in the UTS. Alateyah et al. [26] reported similar behavior for pure Mg. Similar findings were reported by Jin et al. [95], as they achieved an improvement of the AZ31 alloy by 14% after ECAP processing at 225 °C and 250 °C. Meyer et al. [96] and Agnew et al. [97] reported similar improvement in the ductility of AZ31B, whereas they noticed a decrease in the YS while the UTS kept constant. Alateyah et al. [98] generated a comprehensive statistical analysis for the effect of ECAP parameters on the tensile properties of pure Mg. Mostaed et al. [24] achieved improvement in the EL by 30% of ZK60 alloy after ECAP processing through 4-passes at 150 °C. Naik et al. [85] reported a similar trend for AM80 alloys. 

## 4. Conclusions

ZX30 biodegradable Mg alloy (Mg-3Zn-0.6 Zr-0.4 Mn, wt%) billets were annealed for 16 h at a temperature of 430 °C. The AA billets were then subjected to ECAP processing through 1-P, 2-Bc, and 4-Bc at 250 °C. Microstructural evolution, crystallographic texture, electrochemical measurements, and mechanical properties were investigated and analyzed. The following conclusions were drawn:

1. ECAP processing through 4-Bc resulted in a significant grain refinement of 92.8% compared with the AA counterpart.
2. ECAP processing resulted in the evolution of the crystallographic texture of the ZK30 alloy to have the shear plane aligned at 45° relative to the ED.
3. The maximum texture intensity was reduced from 14 times random (AA) to a maximum texture intensity of 8 times random after ECAP processing through 4-Bc.
4. The corrosion rate after processing via 4-Bc was significantly reduced by 99% and 45.25% in the sample with the ringer lactate and 3.5% NaCl fluids, respectively, compared with their AA counterparts.
5. The pitting corrosion resistance of ZK30 improved in ringer lactate by 471.66% and 352% during processing through 1-P and 4-Bc, respectively, compared with the NaCl findings.
6. 1-P processing revealed the best improvement in pitting corrosion resistance in both ringer lactate and NaCl as it increased the pitting corrosion resistance by 3414.4% and 176.76% compared with the AA condition.
7. 4-Bc resulted in improving the HV-values by 86.5% compared with the AA condition.
8. ECAP processing revealed a small improvement in the YS.
9. 4-Bc improved the UTS and EL by 42.8% and 37.2%, respectively, compared with the AA counterpart.

## Figures and Tables

**Table 1:** Grain size, aspect ratio, and grain area data of AA and the ECAPed ZK30 billets.

	Grain Size µm				Grain Aspect Ratio				Grain Area µm^2^			
	AA	1-P	2-Bc	4-Bc	AA	1-P	2-Bc	4-Bc	AA	1-P	2-Bc	4-Bc
Min	3.39	2.24	1.13	0.76	1	1	1	1	9	4.25	1	0.09
Max	76.73	35.22	37.33	17.86	4.77	15.13	6.36	7.5	4624	974	1090	250
Average	26.69	5.43	3.17	1.92	0.46	2.09	1.55	1.69	729	37.14	10.80	3.25
St. Deviation	14.74	4.22	1.92	1.09	1.44	1.39	0.62	0.78	785	84.12	28.13	7.78

**Table 2:** Electrochemical parameters obtained from potentiodynamic polarization curves of the AA and ECAPed ZK30 Mg alloys in ringer lactate and 3.5% NaCl.

	Ringer					NaCl				
	βa (mV.dec^−1^)	-βc (mV.dec^−1^)	Ecorr (V/SCE)	Icorr (µAcm^−2^)	Corrosion Rate (mpy)	βa (mV.dec^−1^)	-βc (mV.dec^−1^)	Ecorr (V/SCE)	Icorr (µAcm^−2^)	Corrosion Rate (mpy)
AA	75.5	137.8	−1.381	596	17.272	76.9	176.6	−1.540	120.40	110.7
1-P	72.8	16.6	−1.361	9.096	0.2654	31.0	145.3	−1.460	84.46	77.6
2-Bc	73.1	220.5	−1.429	44.02	0.404	60.0	169.9	−1.561	102.68	94.1
4-Bc	28.9	82.7	−1.477	5.503	0.1605	76.8	139.9	−1.552	66.03	60.6

**Table 3:** Electrical parameters obtained from fitting the electrochemical impedance spectroscopy (EIS) data of the AA and ECAPed ZK30 Mg alloys in ringer lactate and 3.5% NaCl.

	Ringer					NaCl				
	R_s_ (Ω. cm^2^)	CPE1 (Ω^−1^. s^n^. cm^−2^)	R_L_ (Ω. cm^2^)	R_ct_ (Ω. cm^2^)	L (H. cm^−2^)	R_s_ (Ω. cm^2^)	CPE1 (Ω^−1^. s^n^. cm^−2^)	R_L_ (Ω. cm^2^)	R_ct_ (Ω. cm^2^)	L (H. cm^−2^)
AA	75.5	137.8	−1.381	596	17.272	76.9	176.6	−1.540	120.40	110.7
1-P	72.8	16.6	−1.361	9.096	0.2654	31.0	145.3	−1.460	84.46	77.6
2-Bc	73.1	220.5	−1.429	44.02	0.404	60.0	169.9	−1.561	102.68	94.1
4-Bc	28.9	82.7	−1.477	5.503	0.1605	76.8	139.9	−1.552	66.03	60.6

**Table 4:** Hardness distribution along the LS and TS and the tensile properties of ZK30 billets before and after ECAP processing.

	HV				Yield Stress (MPa)	Ultimate Strength (MPa)	Elongation (EL%)
	LS		TS				
	CR	PR	CR	PR			
AA	52 ± 1		52 ± 1		80 ± 1	238 ± 1	20.4 ± 0.25
1-P	72 ± 1	83 ± 0.5	76 ± 1	82 ± 1.5	86 ± 1	304 ± 3	37 ± 2
2-Bc	81 ± 1	86 ± 1	80 ± 1	84 ± 0.5	88 ± 2	315 ± 2	36.6 ± 1.5
4-Bc	90 ± 0.5	97 ± 0.5	87 ± 0.5	93 ± 1	96 ± 2	340 ± 2	28 ± 1

## Data Availability

All the raw data supporting the conclusion of this paper were provided by the authors.
